# Supplementary material for: Evolution of Gene Expression across Species and Specialized Zooids in Siphonophora
Source: Mol Biol Evol. 2022 Feb 3;39(2):msac027. doi: 10.1093/molbev/msac027 (PMC8844502; doi:10.1093/molbev/msac027)
Supplement: msac027_Supplementary_Data [file msac027_supplementary_data.zip › Github_repo_Siphonophore_Expression-paper_v1.zip!/Github_repo_Siphonophore_Expression-paper_v1/figures/plot_pca_ortholog2.pdf]

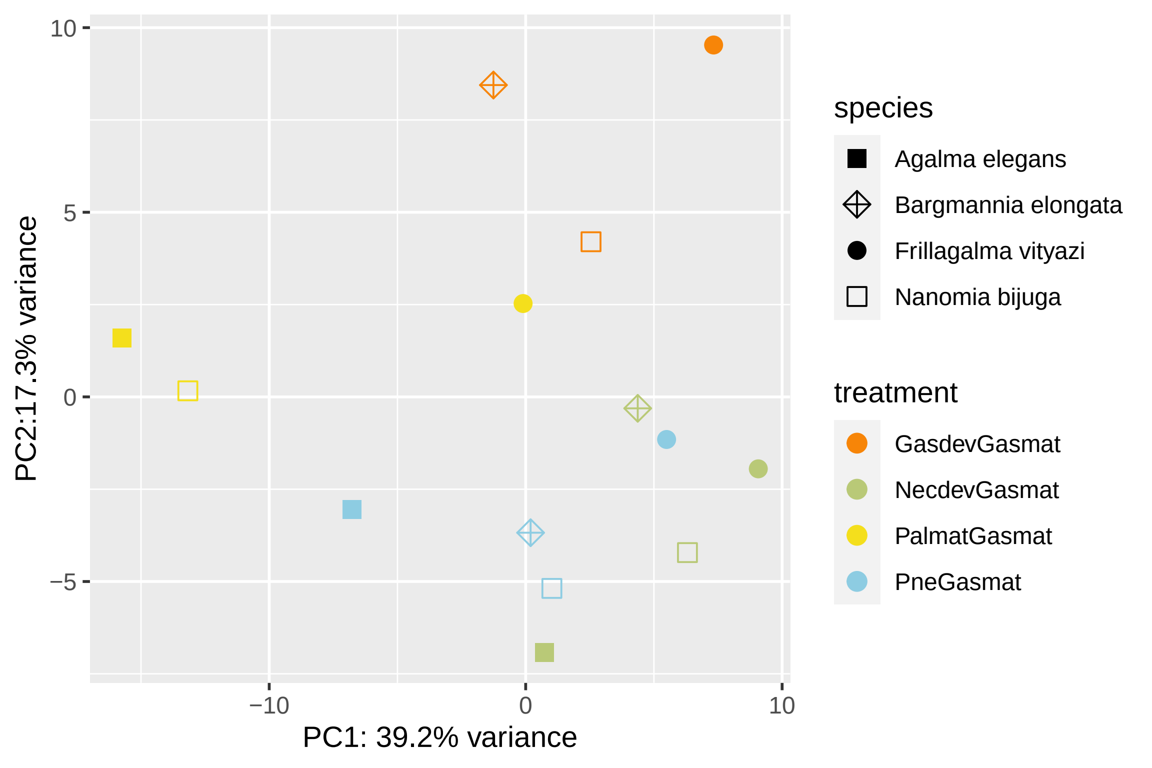

PC2:17.3% variance
PC1: 39.2% variance
10
5
0
−5
−10
0
10
species
Agalma elegans
Bargmannia elongata
Frillagalma vityazi
Nanomia bijuga
treatment
GasdevGasmat
NecdevGasmat
PalmatGasmat
PneGasmat
