# Supplementary material for: Evolution of Gene Expression across Species and Specialized Zooids in Siphonophora
Source: Mol Biol Evol. 2022 Feb 3;39(2):msac027. doi: 10.1093/molbev/msac027 (PMC8844502; doi:10.1093/molbev/msac027)
Supplement: msac027_Supplementary_Data [file msac027_supplementary_data.zip › Github_repo_Siphonophore_Expression-paper_v1.zip!/Github_repo_Siphonophore_Expression-paper_v1/figures/DGE_figure.pdf]

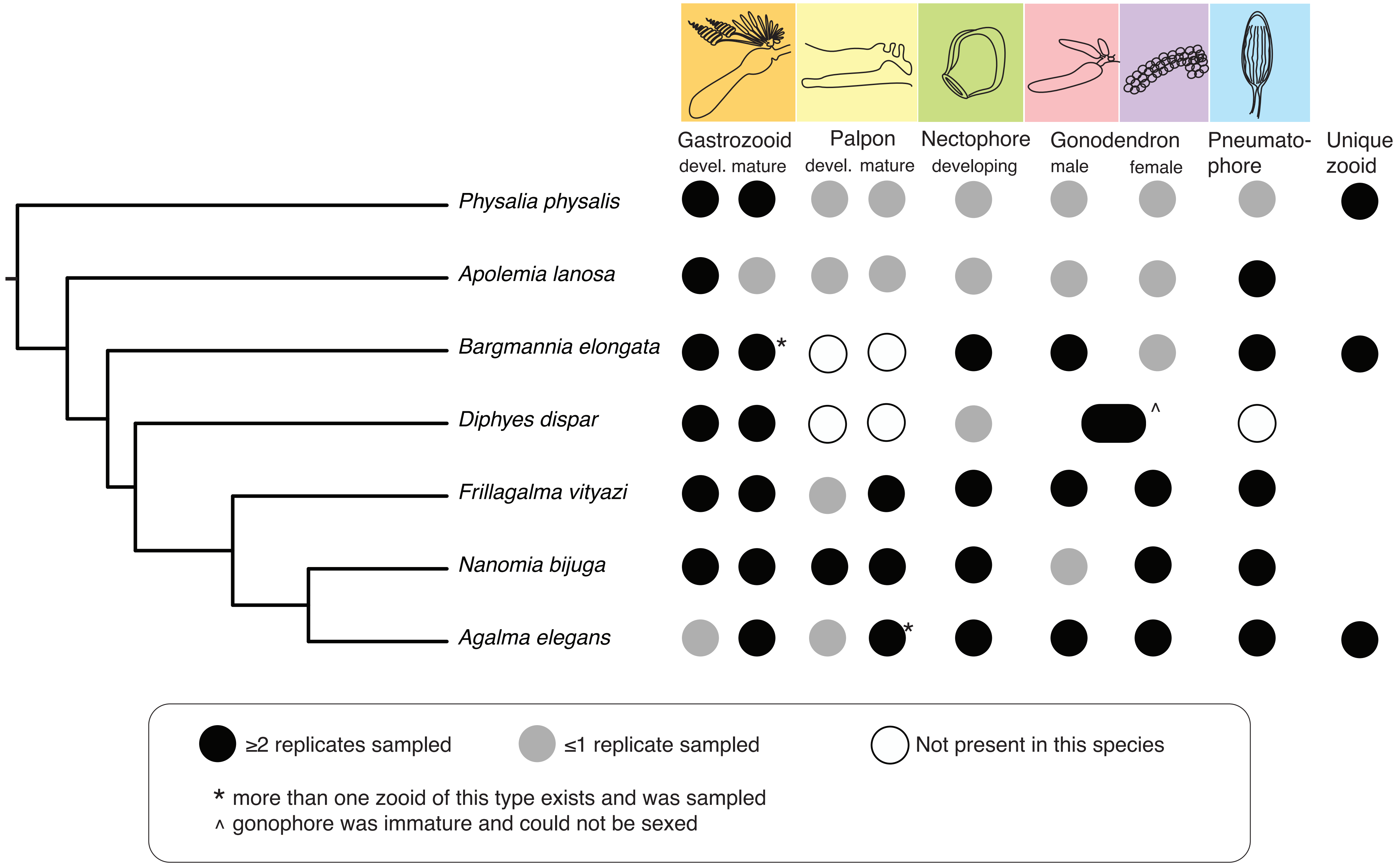
Gastrozooid
devel. mature
Palpon
devel. mature
Nectophore
developing
Gonodendron
male female
Pneumato-
phore
Unique
zooid
Physalia physalis
Apolemia lanosa
Bargmannia elongata
Diphyes dispar
Frillagalma vityazi
Nanomia bijuga
Agalma elegans
*
*
^
≥2 replicates sampled
≤1 replicate sampled
Not present in this species
* more than one zooid of this type exists and was sampled
^ gonophore was immature and could not be sexed
