# Supplementary material for: Evolution of Gene Expression across Species and Specialized Zooids in Siphonophora
Source: Mol Biol Evol. 2022 Feb 3;39(2):msac027. doi: 10.1093/molbev/msac027 (PMC8844502; doi:10.1093/molbev/msac027)
Supplement: msac027_Supplementary_Data [file msac027_supplementary_data.zip › Github_repo_Siphonophore_Expression-paper_v1.zip!/Github_repo_Siphonophore_Expression-paper_v1/figures/Variance_explained2.pdf]

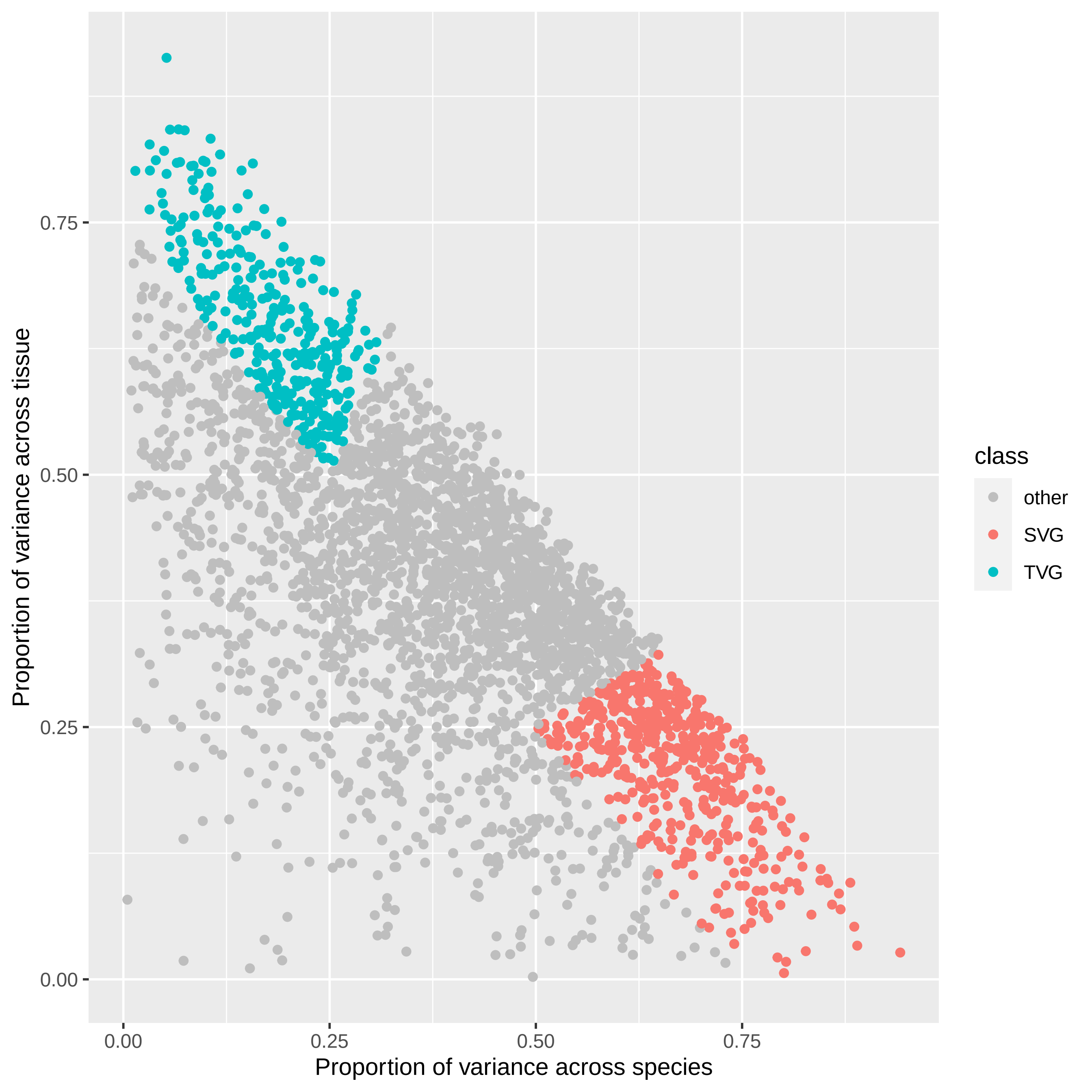

Proportion of variance across tissue
0.75
0.50
0.25
0.00
0.00
0.25
0.50
0.75
Proportion of variance across species
class
other
SVG
TVG
