# Supplementary material for: Evolution of Gene Expression across Species and Specialized Zooids in Siphonophora
Source: Mol Biol Evol. 2022 Feb 3;39(2):msac027. doi: 10.1093/molbev/msac027 (PMC8844502; doi:10.1093/molbev/msac027)
Supplement: msac027_Supplementary_Data [file msac027_supplementary_data.zip › Github_repo_Siphonophore_Expression-paper_v1.zip!/Github_repo_Siphonophore_Expression-paper_v1/figures/plot_pca_ortholog.pdf]

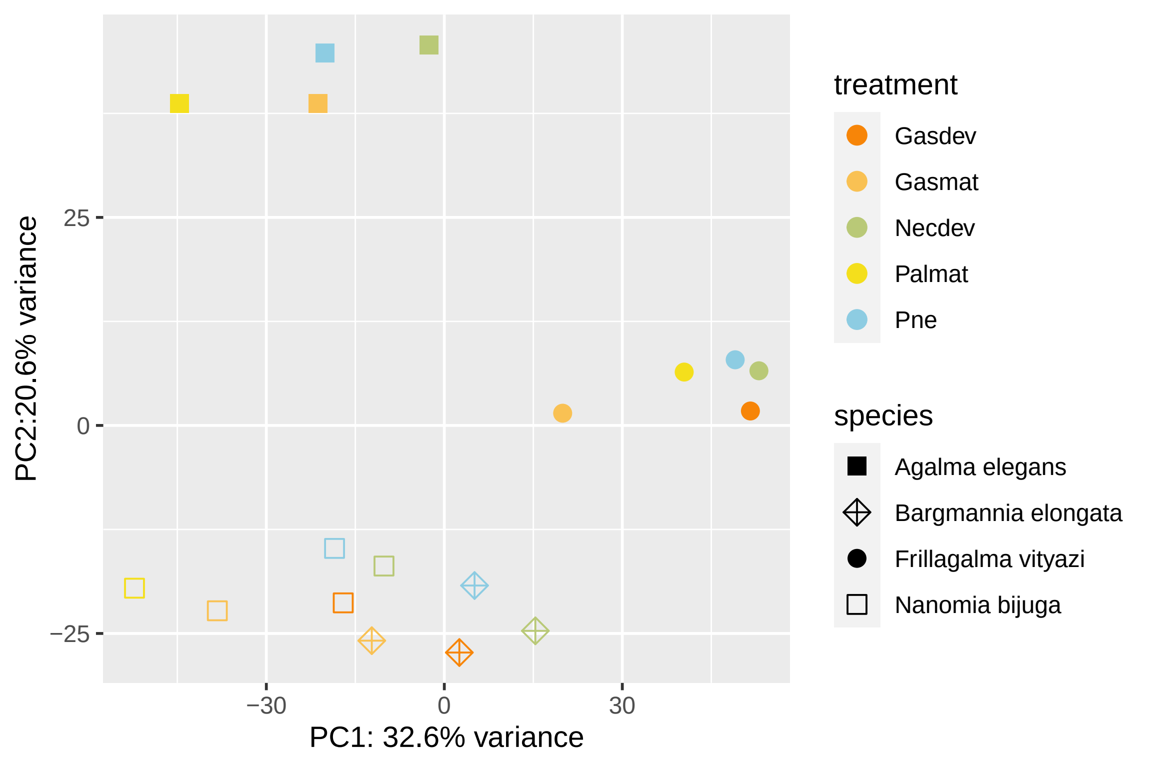

PC2:20.6% variance
25
0
−25
−30
0
30
PC1: 32.6% variance
treatment
Gasdev
Gasmat
Necdev
Palmat
Pne
species
Agalma elegans
Bargmannia elongata
Frillagalma vityazi
Nanomia bijuga
