# Supplementary material for: Evolution of Gene Expression across Species and Specialized Zooids in Siphonophora
Source: Mol Biol Evol. 2022 Feb 3;39(2):msac027. doi: 10.1093/molbev/msac027 (PMC8844502; doi:10.1093/molbev/msac027)
Supplement: msac027_Supplementary_Data [file msac027_supplementary_data.zip › Github_repo_Siphonophore_Expression-paper_v1.zip!/Github_repo_Siphonophore_Expression-paper_v1/figures/Methods_figure.pdf]

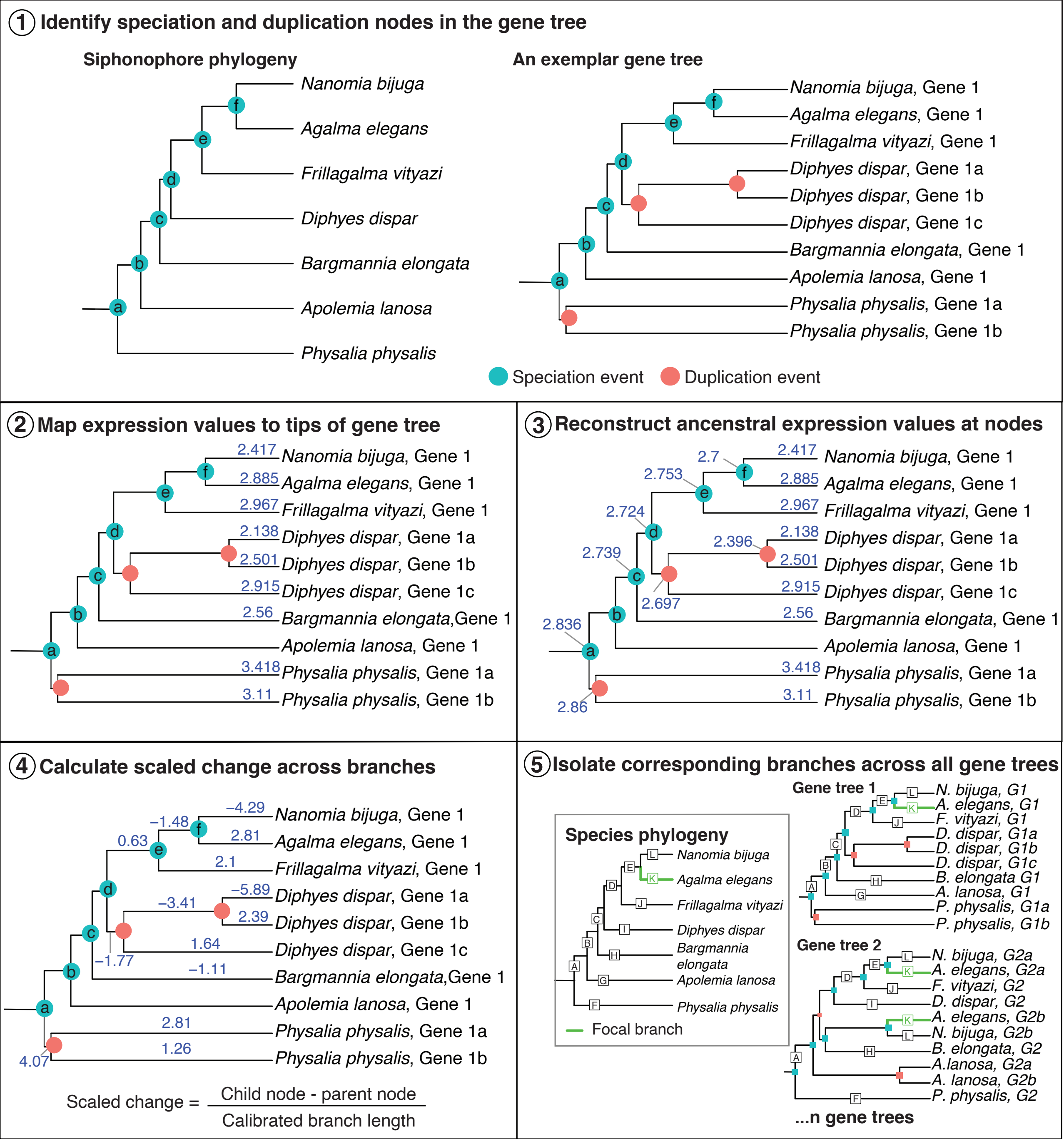

1 Identify speciation and duplication nodes in the gene tree
Siphonophore phylogeny
Nanomia bijuga
Agalma elegans
Frillagalma vityazi
Diphyes dispar
Bargmannia elongata
Apolemia lanosa
Physalia physalis
An exemplar gene tree
Nanomia bijuga, Gene 1
Agalma elegans, Gene 1
Frillagalma vityazi, Gene 1
Diphyes dispar, Gene 1a
Diphyes dispar, Gene 1b
Diphyes dispar, Gene 1c
Bargmannia elongata, Gene 1
Apolemia lanosa, Gene 1
Physalia physalis, Gene 1a
Physalia physalis, Gene 1b
Speciation event
Duplication event
2 Map expression values to tips of gene tree
2.417 Nanomia bijuga, Gene 1
2.885 Agalma elegans, Gene 1
2.967 Frillagalma vityazi, Gene 1
2.138 Diphyes dispar, Gene 1a
2.501 Diphyes dispar, Gene 1b
2.915 Diphyes dispar, Gene 1c
2.56 Bargmannia elongata,Gene 1
Apolemia lanosa, Gene 1
3.418 Physalia physalis, Gene 1a
3.11 Physalia physalis, Gene 1b
3 Reconstruct ancenstral expression values at nodes
2.7
2.753
2.724
2.739
2.396
2.697
2.836
2.86
2.417 Nanomia bijuga, Gene 1
2.885 Agalma elegans, Gene 1
2.967 Frillagalma vityazi, Gene 1
2.138 Diphyes dispar, Gene 1a
2.501 Diphyes dispar, Gene 1b
2.915 Diphyes dispar, Gene 1c
2.56 Bargmannia elongata, Gene 1
Apolemia lanosa, Gene 1
3.418 Physalia physalis, Gene 1a
3.11 Physalia physalis, Gene 1b
4 Calculate scaled change across branches
−4.29 Nanomia bijuga, Gene 1
−1.48
0.63
2.81 Agalma elegans, Gene 1
2.1 Frillagalma vityazi, Gene 1
−5.89 Diphyes dispar, Gene 1a
−3.41
2.39 Diphyes dispar, Gene 1b
1.64 Diphyes dispar, Gene 1c
−1.77
−1.11 Bargmannia elongata,Gene 1
Apolemia lanosa, Gene 1
2.81 Physalia physalis, Gene 1a
1.26 Physalia physalis, Gene 1b
4.07
Scaled change = Child node - parent node / Calibrated branch length
5 Isolate corresponding branches across all gene trees
Species phylogeny
Nanomia bijuga
Agalma elegans
Frillagalma vityazi
Diphyes dispar
Bargmannia elongata
Apolemia lanosa
Physalia physalis
Focal branch
Gene tree 1
N. bijuga, G1
A. elegans, G1
F. vityazi, G1
D. dispar, G1a
D. dispar, G1b
D. dispar, G1c
B. elongata G1
A. lanosa, G1
P. physalis, G1a
P. physalis, G1b
Gene tree 2
N. bijuga, G2a
A. elegans, G2a
F. vityazi, G2
D. dispar, G2
A. elegans, G2b
N. bijuga, G2b
B. elongata, G2
A.lanosa, G2a
A. lanosa, G2b
P. physalis, G2
...n gene trees
