# Supplementary material for: Evolution of Gene Expression across Species and Specialized Zooids in Siphonophora
Source: Mol Biol Evol. 2022 Feb 3;39(2):msac027. doi: 10.1093/molbev/msac027 (PMC8844502; doi:10.1093/molbev/msac027)
Supplement: msac027_Supplementary_Data [file msac027_supplementary_data.zip › Github_repo_Siphonophore_Expression-paper_v1.zip!/Github_repo_Siphonophore_Expression-paper_v1/figures/Methods_DGE.pdf]

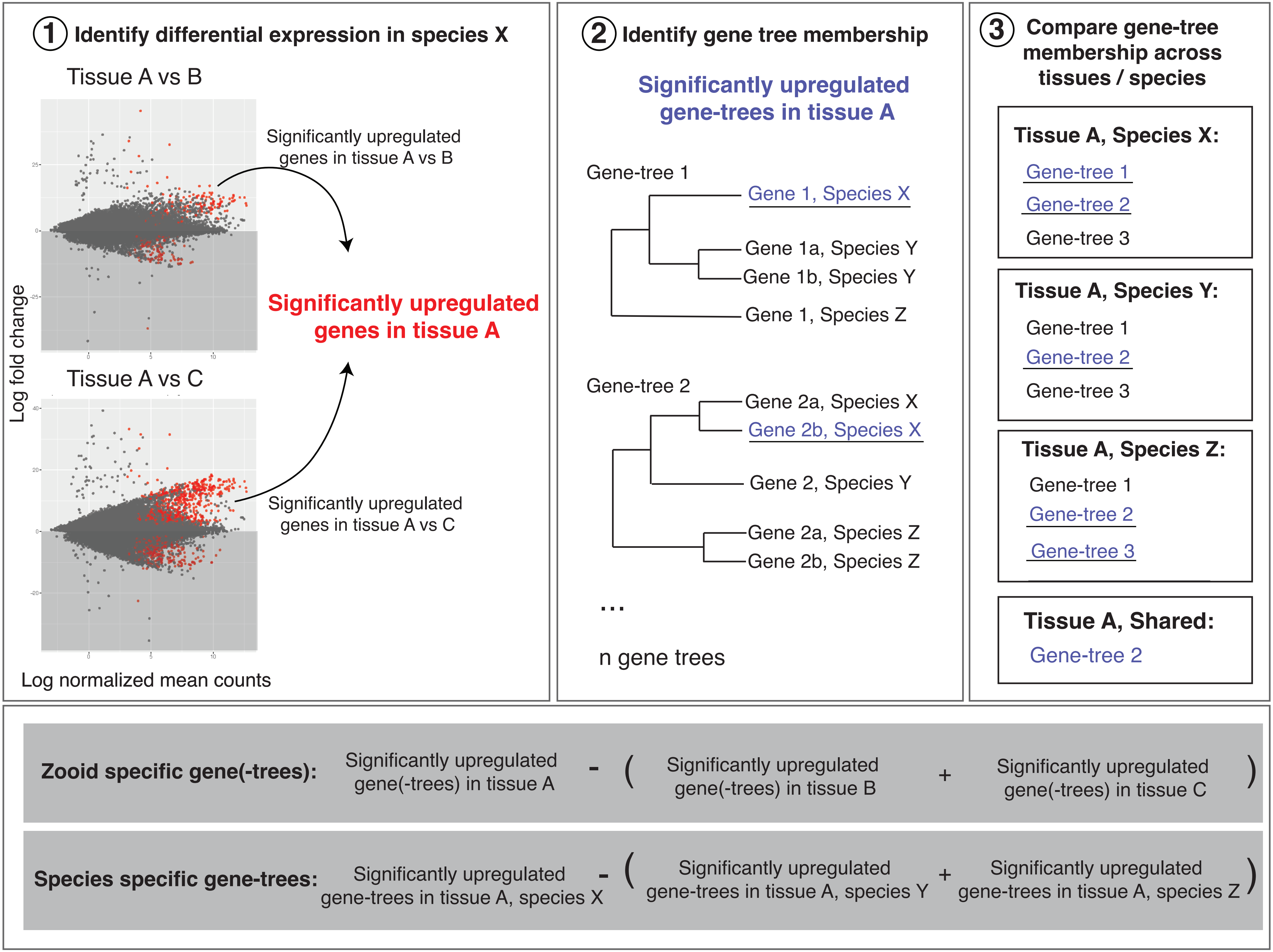
1 Identify differential expression in species X
Tissue A vs B
Significantly upregulated genes in tissue A vs B
Significantly upregulated genes in tissue A
Tissue A vs C
Significantly upregulated genes in tissue A vs C
Log fold change
Log normalized mean counts
2 Identify gene tree membership
Significantly upregulated gene-trees in tissue A
Gene-tree 1
Gene 1, Species X
Gene 1a, Species Y
Gene 1b, Species Y
Gene 1, Species Z
Gene-tree 2
Gene 2a, Species X
Gene 2b, Species X
Gene 2, Species Y
Gene 2a, Species Z
Gene 2b, Species Z
...
n gene trees
3 Compare gene-tree membership across tissues / species
Tissue A, Species X:
Gene-tree 1
Gene-tree 2
Gene-tree 3
Tissue A, Species Y:
Gene-tree 1
Gene-tree 2
Gene-tree 3
Tissue A, Species Z:
Gene-tree 1
Gene-tree 2
Gene-tree 3
Tissue A, Shared:
Gene-tree 2
Zooid specific gene(-trees): Significantly upregulated gene(-trees) in tissue A - (Significantly upregulated gene(-trees) in tissue B + Significantly upregulated gene(-trees) in tissue C)
Species specific gene-trees: Significantly upregulated gene-trees in tissue A, species X - (Significantly upregulated gene-trees in tissue A, species Y + Significantly upregulated gene-trees in tissue A, species Z)
