# Supplementary material for: Evolution of Gene Expression across Species and Specialized Zooids in Siphonophora
Source: Mol Biol Evol. 2022 Feb 3;39(2):msac027. doi: 10.1093/molbev/msac027 (PMC8844502; doi:10.1093/molbev/msac027)
Supplement: msac027_Supplementary_Data [file msac027_supplementary_data.zip › Github_repo_Siphonophore_Expression-paper_v1.zip!/Github_repo_Siphonophore_Expression-paper_v1/figures/Wnt_plot2_new.pdf]

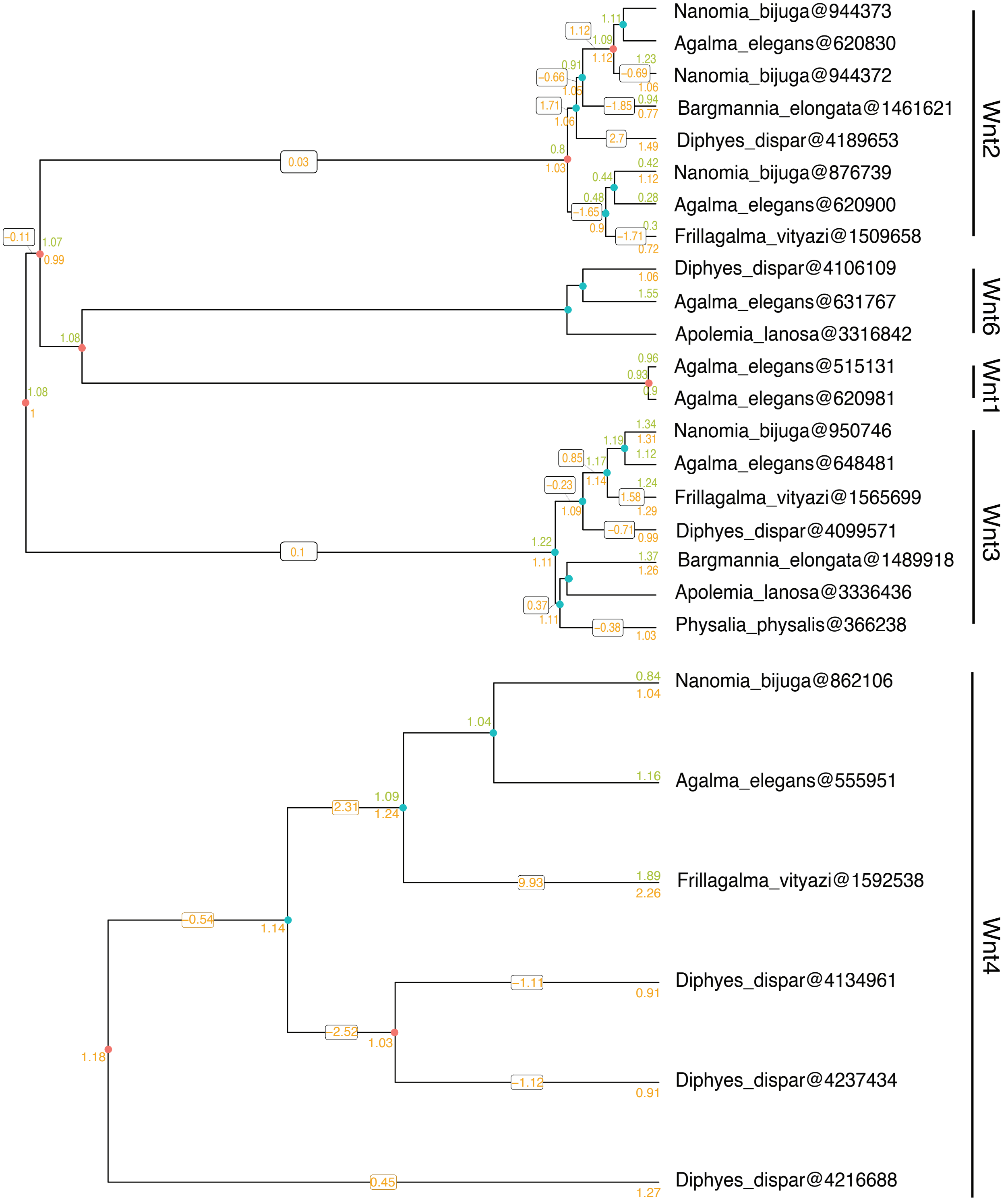
Nanomia_bijuga@944373
Agalma_elegans@620830
Nanomia_bijuga@944372
Bargmannia_elongata@1461621
Diphyes_dispar@4189653
Nanomia_bijuga@876739
Agalma_elegans@620900
Frillagalma_vityazi@1509658
Diphyes_dispar@4106109
Agalma_elegans@631767
Apolemia_lanosa@3316842
Agalma_elegans@515131
Agalma_elegans@620981
Nanomia_bijuga@950746
Agalma_elegans@648481
Frillagalma_vityazi@1565699
Diphyes_dispar@4099571
Bargmannia_elongata@1489918
Apolemia_lanosa@3336436
Physalia_physalis@366238
Nanomia_bijuga@862106
Agalma_elegans@555951
Frillagalma_vityazi@1592538
Diphyes_dispar@4134961
Diphyes_dispar@4237434
Diphyes_dispar@4216688
Wnt2
Wnt6
Wnt1
Wnt3
Wnt4
