# Supplementary material for: Evolution of Gene Expression across Species and Specialized Zooids in Siphonophora
Source: Mol Biol Evol. 2022 Feb 3;39(2):msac027. doi: 10.1093/molbev/msac027 (PMC8844502; doi:10.1093/molbev/msac027)
Supplement: msac027_Supplementary_Data [file msac027_supplementary_data.zip › Github_repo_Siphonophore_Expression-paper_v1.zip!/Github_repo_Siphonophore_Expression-paper_v1/figures/Siphonophoresare.pdf]

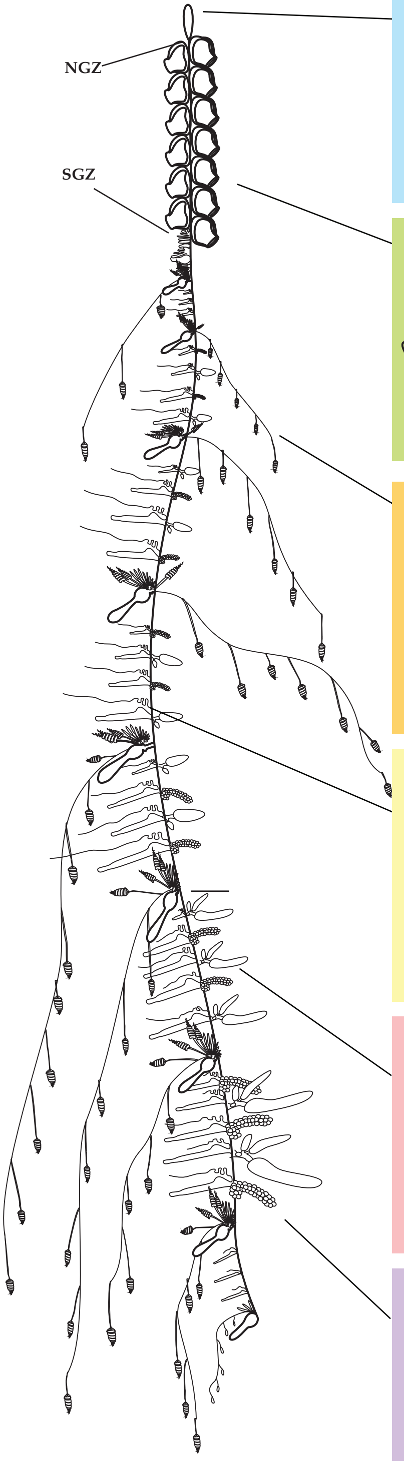

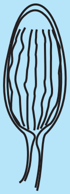

Pneumatophore

Tissue specialized in buoyancy, flotation, and geotaxis. Thought to generate carbon monoxide. Not a zooid.

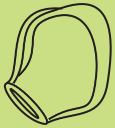

Nectophore

Zooid specialized for swimming. Homologous to medusae. Highly muscular and propels the colony via contractions that generate water jets out of the ostium

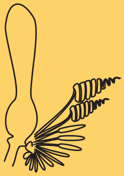

Gastrozooid

Zooid specialized for feeding. Homologous to polyps. Ingests food, and extracellularly digests prey. Typically has a tentacle for prey capture. Has a nematogenic region that supplies nematocysts to the tentacle.

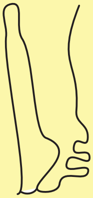

Palpon

Zooid specialized for feeding. Derived gastrozooid. Considered to be an accessory digestive zooid. Hypothesized to play defensive and sensory functions. Typically has a reduced tentacle (palpacle).

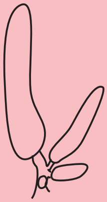

Male gonodendron

Compound structures with multiple **gonophores** (zooids homologous to medusae) that contain male gametes and are the site of spermatogenesis.

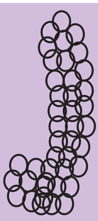

Female gonodendron

Compound structures with multiple **gonophores** (zooids homologous to medusae) that contain female gametes and are the site of oogenesis.
