# Supplementary material for: Evolution of Gene Expression across Species and Specialized Zooids in Siphonophora
Source: Mol Biol Evol. 2022 Feb 3;39(2):msac027. doi: 10.1093/molbev/msac027 (PMC8844502; doi:10.1093/molbev/msac027)
Supplement: msac027_Supplementary_Data [file msac027_supplementary_data.zip › Github_repo_Siphonophore_Expression-paper_v1.zip!/Github_repo_Siphonophore_Expression-paper_v1/figures/technical-replicate.pdf]

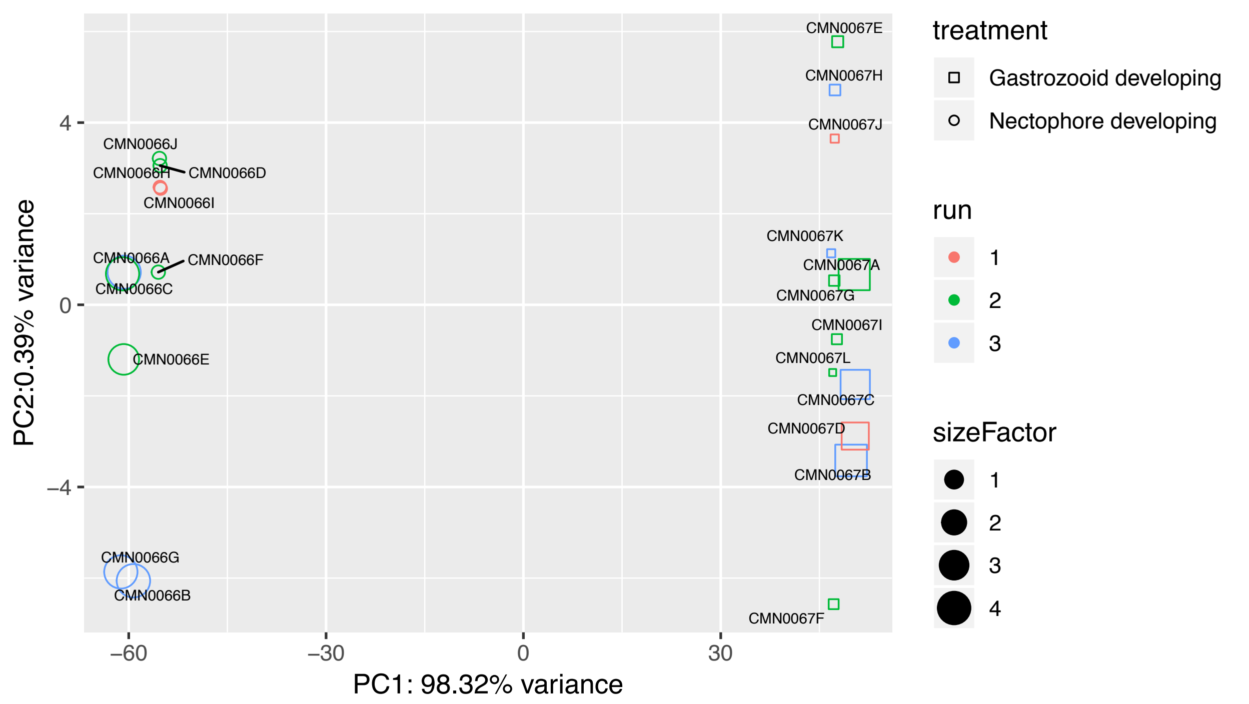

CMN0067E
CMN0067H
CMN0067J
CMN0066J
CMN0066H
CMN0066D
CMN0066I
CMN0067K
CMN0066A
CMN0066F
CMN0067A
CMN0066C
CMN0067G
CMN0067I
CMN0066E
CMN0067L
CMN0067C
CMN0067D
CMN0067B
CMN0066G
CMN0066B
CMN0067F
4
0
−4
−60
−30
0
30
PC1: 98.32% variance
PC2:0.39% variance
treatment
Gastrozooid developing
Nectophore developing
run
1
2
3
sizeFactor
1
2
3
4
