# Supplementary material for: Evolution of Gene Expression across Species and Specialized Zooids in Siphonophora
Source: Mol Biol Evol. 2022 Feb 3;39(2):msac027. doi: 10.1093/molbev/msac027 (PMC8844502; doi:10.1093/molbev/msac027)
Supplement: msac027_Supplementary_Data [file msac027_supplementary_data.zip › Github_repo_Siphonophore_Expression-paper_v1.zip!/Github_repo_Siphonophore_Expression-paper_v1/figures/Toxin_plot.pdf]

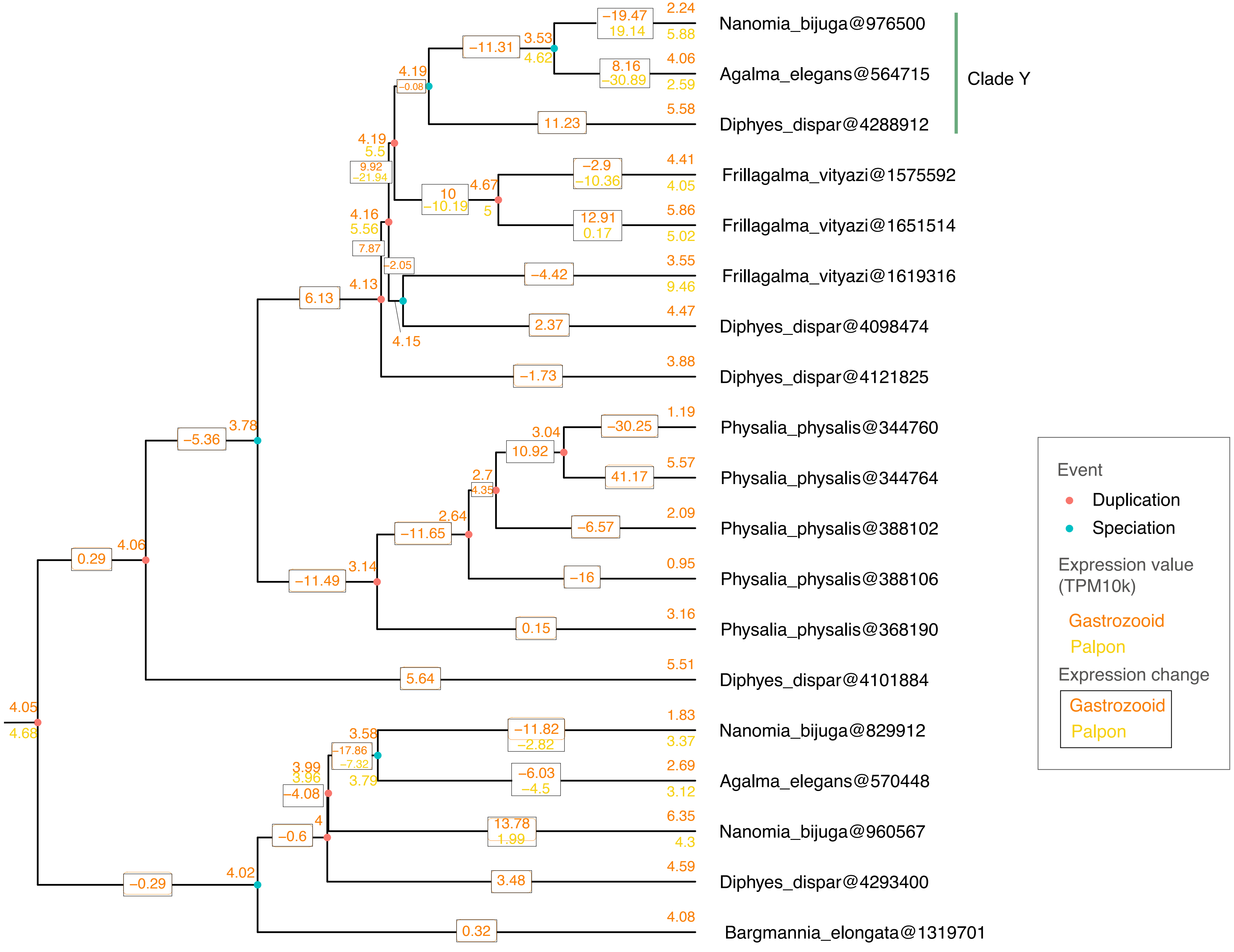
Nanomia_bijuga@976500
Agalma_elegans@564715
Diphyes_dispar@4288912
Frillagalma_vityazi@1575592
Frillagalma_vityazi@1651514
Frillagalma_vityazi@1619316
Diphyes_dispar@4098474
Diphyes_dispar@4121825
Physalia_physalis@344760
Physalia_physalis@344764
Physalia_physalis@388102
Physalia_physalis@388106
Physalia_physalis@368190
Diphyes_dispar@4101884
Nanomia_bijuga@829912
Agalma_elegans@570448
Nanomia_bijuga@960567
Diphyes_dispar@4293400
Bargmannia_elongata@1319701
Clade Y
Event
Duplication
Speciation
Expression value (TPM10k)
Gastrozooid
Palpon
Expression change
Gastrozooid
Palpon
