# Supplementary material for: Evolution of Gene Expression across Species and Specialized Zooids in Siphonophora
Source: Mol Biol Evol. 2022 Feb 3;39(2):msac027. doi: 10.1093/molbev/msac027 (PMC8844502; doi:10.1093/molbev/msac027)
Supplement: msac027_Supplementary_Data [file msac027_supplementary_data.zip › Github_repo_Siphonophore_Expression-paper_v1.zip!/Github_repo_Siphonophore_Expression-paper_v1/figures/changes.pdf]

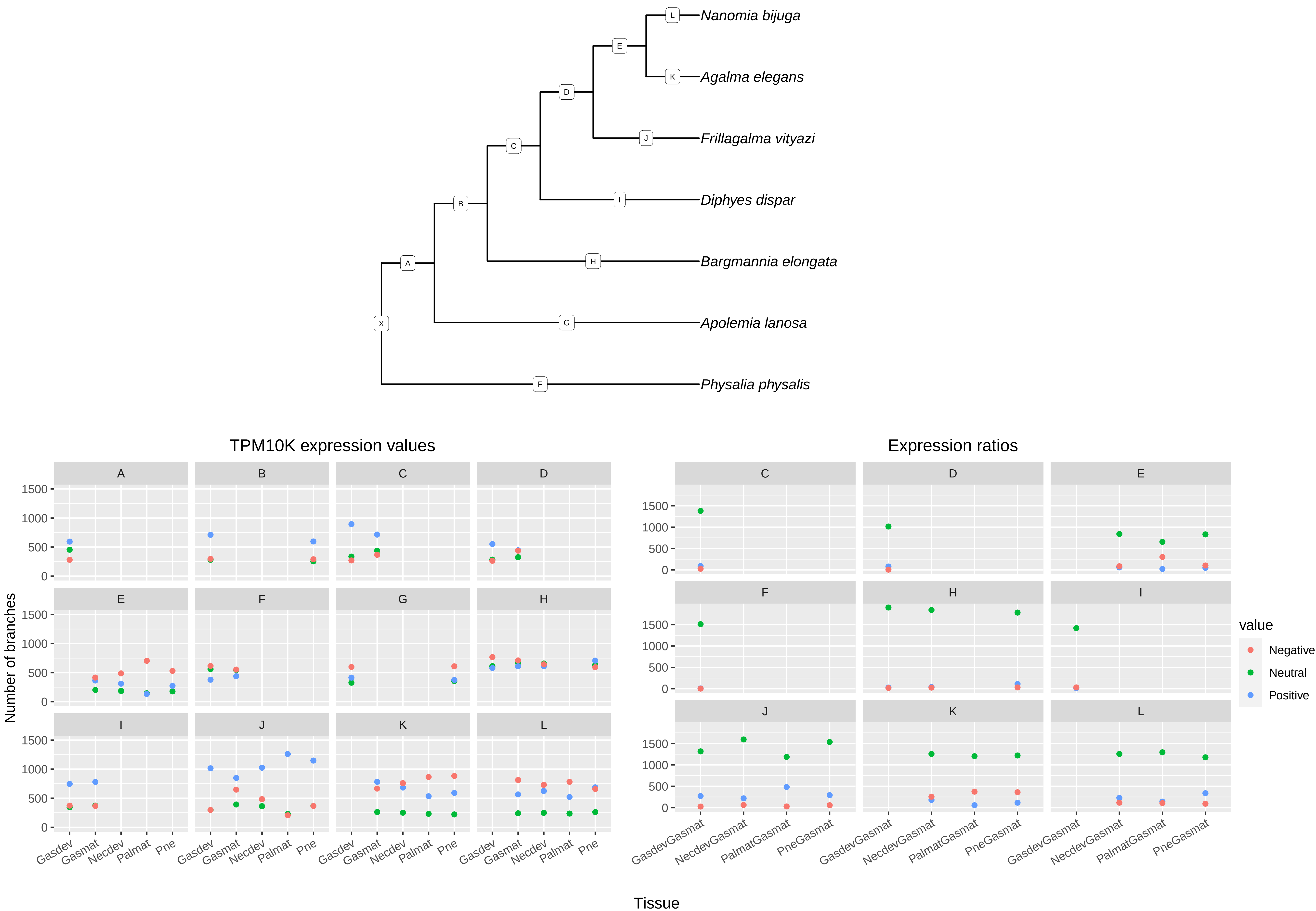

Nanomia bijuga
Agalma elegans
Frillagalma vityazi
Diphyes dispar
Bargmannia elongata
Apolemia lanosa
Physalia physalis
L
K
E
D
J
C
I
B
H
A
G
X
F
TPM10K expression values
Expression ratios
Number of branches
Tissue
Gasdev
Gasmat
Necdev
Palmat
Pne
GasdevGasmat
NecdevGasmat
PalmatGasmat
PneGasmat
0
500
1000
1500
value
Negative
Neutral
Positive
